# Resilience of people with a history of mental disorder during the COVID-19 pandemic: a 2-years longitudinal prospective study

**DOI:** 10.1192/j.eurpsy.2023.249

**Published:** 2023-07-19

**Authors:** I. Pinucci, L. Tarsitani, F. Tedeschi, M. Patanè, R. Serra, D. Papola, C. Palantza, C. Acartürk, R. Bryant, S. Burchert, D. Fuhr, B. J. Hall, E. Mittendorfer-Rutz, N. Morina, C. Panter-Brick, S. Quero, S. Seedat, H. Setyowibowo, J. van der Waerden, M. Pasquini, M. Sijbrandij, C. Barbui

**Affiliations:** 1 Department of Human Neurosciences, Sapienza University of Rome, Rome, Italy; 2 Department of Clinical, Neuro-, and Developmental Psychology and WHO Collaborating Center for Research and Dissemination of Psychological Interventions, Vrije Universiteit, Amsterdam, Netherlands; 3 WHO Collaborating Centre for Research and Training in Mental Health and Service Evaluation, Department of Neuroscience, Biomedicine and Movement Sciences, University of Verona, Verona, Italy; 4 Department of Psychology, Koc University, İstanbul, Türkiye; 5 School of Psychology, University of New South Wales, Sydney, Australia; 6 Division of Clinical Psychological Intervention, Department of Education and Psychology, Freie Universität Berlin, Berlin; 7 Leibniz Institute for Prevention Research and Epidemiology; 8 Health Sciences, University of Bremen, Bremen, Germany; 9 Center for Global Health Equity, New York University Shanghai, Shanghai, China; 10 Department of Clinical Neuroscience, Division of Insurance Medicine, Karolinska Institutet, Stockholm, Sweden; 11 Department of Consultation-Liaison Psychiatry and Psychosomatic Medicine, University Hospital of Zurich, University of Zurich, Zurich, Switzerland; 12 Department of Anthropology, Yale University, New Haven, United States; 13 Department of Basic, Clinical Psychology and Psychobiology, Universitat Jaume I, Castellón; 14 CIBER de Fisiopatología de la Obesidad y Nutrición (CIBEROBN), Carlos III Institute of Health, Madrid, Spain; 15 Department of Psychiatry, Faculty of Medcine and Health Sciences, Stellenbosch University, Cape Town, South Africa; 16 Department of Educational Psychology, Faculty of Psychology, Universitas Padjadjaran, Jatinangor, Indonesia; 17 INSERM, Institut Pierre Louis d’Epidémiologie et de Santé Publique/ERES, Sorbonne Université, Paris, France

## Abstract

**Introduction:**

During the COVID-19 pandemic, people with mental disorders were exposed to a common and prolonged source of stress. Studies focusing on the consequences of the pandemic on individuals with a history of mental disorder are scarce, but they suggest a higher vulnerability as compared to the general population.

**Objectives:**

We aimed at identifying predictors of stress resilience maintained over time among these people during the first two years of the pandemic.

**Methods:**

The presented study is part of a larger 2-year, 5-wave international longitudinal online survey.

The Patient Health Questionnaire, the Generalized Anxiety Disorder scale and the PTSD Checklist DSM-5 were used as latent class indicators for a proxy measure of distress. Specifically, a Latent-Class Analysis was performed to identify a group that showed resilient outcomes across all waves.

We investigated socio-demographic characteristics, economic and housing status, lifestyle and habits, pandemic-related issues, and chronic disease. Adherence to and approval of the restrictions imposed, trust in governments and the scientific community during the pandemic were also assessed. Social support, fear of contamination and personal values were investigated respectively through the Oslo Social Support Scale, the Padua Inventory, and the Portrait Values Questionnaire. The aforementioned characteristics were used to predict sustained resilience through a logistic regression.

**Results:**

A total of 1711 participants out of the total sample (8011 participants from 13 different countries) reported a diagnosis of mental disorder before the pandemic. Nine hundred forty-three participants completed at least three of the five versions of the survey and were included in the analysis. A latent class of participants with resilience maintained over time (*sustained resilience*) was identified, with an estimated probability of 24.8%. The demographic and clinical variables associated with a higher chance of *sustained resilience* were older age, maintaining a job during the pandemic, and having a larger number of people in the household. In contrast, female gender, losing job during the pandemic, having difficulty meeting basic needs, greater fear of contamination, a stronger focus on hedonism, less social support and feeling lonely resulted in a lower likelihood of being *sustained resilient.*

**Conclusions:**

This study identified a number of factors that may help predict resilient outcomes maintained over time in people with mental disorders. COVID-19 related predictors of *sustained resilience* are new findings which might inform resilience-building interventions during pandemics.

**Disclosure of Interest:**

None Declared

